# Mitochondrial Thioredoxin-Glutathione Reductase from Larval *Taenia crassiceps* (Cysticerci)

**DOI:** 10.1155/2010/719856

**Published:** 2010-06-22

**Authors:** Alberto Guevara-Flores, Irene P. del Arenal, Guillermo Mendoza-Hernández, Juan Pablo Pardo, Oscar Flores-Herrera, Juan L. Rendón

**Affiliations:** Departamento de Bioquímica, Facultad de Medicina, Universidad Nacional Autónoma de México, Apartado Postal no. 70-159, 04510 México, DF, Mexico

## Abstract

Mitochondrial thioredoxin-glutathione reductase was purified from larval *Taenia crassiceps* (cysticerci). The preparation showed NADPH-dependent reductase activity with either thioredoxin or GSSG, and was able to perform thiol/disulfide exchange reactions. At 25°C specific activities were 437 ± 27 mU mg^−1^ and 840 ± 49 mU mg^−1^ with thioredoxin and GSSG, respectively. Apparent K_m_ values were 0.87 ± 0.04 *μ*M, 41 ± 6 *μ*M and 19 ± 10 *μ*M for thioredoxin, GSSG and NADPH, respectively. Thioredoxin from eukaryotic sources was accepted as substrate. The enzyme reduced H_2_O_2_ in a NADPH-dependent manner, although with low catalytic efficiency. In the presence of thioredoxin, mitochondrial TGR showed a thioredoxin peroxidase-like activity. All disulfide reductase activities were inhibited by auranofin, suggesting mTGR is dependent on selenocysteine. The reductase activity with GSSG showed a higher dependence on temperature as compared with the DTNB reductase activity. The variation of the GSSG- and DTNB reductase activities on pH was dependent on the disulfide substrate. Like the cytosolic isoform, mTGR showed a hysteretic kinetic behavior at moderate or high GSSG concentrations, but it was less sensitive to calcium. The enzyme was able to protect glutamine synthetase from oxidative inactivation, suggesting that mTGR is competent to contend with oxidative stress.

## 1. Introduction

The mitochondrion is a cell organelle where much of the reactive oxygen species (ROS) are produced, mainly as collateral reactions of the respiratory complexes [1]. An excessive increase in the concentration of such species will result in severe oxidative stress [2]. However, cells have the ability to cope with the endogenously generated ROS through both enzymatic and nonenzymatic defense systems [3]. Additionally, there are other antioxidant protection systems with the ability to revert the damage produced by ROS. Such is the case of the reversible thiol-disulfide exchange reactions, which are involved in the maintenance of a proper redox environment in cells. Outstanding in this sense are the glutathione and thioredoxin systems, with a broad distribution in the living world and playing a variety of physiological functions [4, 5]. In both cases, the proper operation as antioxidants depends on the reduced state of the molecule. For the majority of the organisms, independent and specific NADPH-dependent disulfide reductases are present. Thus, while glutathione reductase (GR) is involved in the reduction of GSSG, thioredoxin reductase (TrxR) reduces oxidized thioredoxin. Both enzymes are homodimeric flavoproteins members of the disulfide oxidoreductase family [6]. In the specific case of TrxR, two different major forms are known. The enzyme is represented by a dimeric protein constituted by subunits of about 35 kDa in bacteria, plants, and some unicellular eukaryotes, [7]. In mammals, TrxR exists as a dimeric protein formed by subunits of about 55 kDa [7]. Furthermore, the mammalian enzyme is dependent on a selenocysteine residue for activity, located at its carboxyl end [8]. In these vertebrates, both cytosolic and mitochondrial isoforms of TrxR have been identified [9, 10].

Recently, the existence of an interesting isoform of TrxR, named thioredoxin-glutathione reductase (TGR), was reported in adult mammalian testes [11]. Such variant arose from the fusion of the classical mammalian TrxR module with a glutaredoxin-like domain at its N-terminal end. Like mammalian TrxR, the enzymatic activity of TGR is also dependent on selenocysteine. Interestingly, TGR is a multifunctional enzyme, because it has the ability to reduce both GSSG and Trx, and to catalyze thiol/disulfide exchanges, a property dependent on the presence of the glutaredoxin domain [11]. The presence of TGR has been demonstrated in a variety of vertebrates [12], as well as in the parasitic representatives of the flatworms [13–15]. It is worth to note that in the latter, typical GR or TrxR are absent, and the reduction of both GSSG and oxidized Trx is carried out by TGR. Thus, in these organisms the antioxidant GSH- and Trx-dependent systems are based on a single reductase. Because *Taenia crassiceps* mitochondria have the ability to produce H_2_O_2_ in significant amounts [16], antioxidant protective systems must be present in this organelle to avoid the development of endogenous oxidative stress. However, no information on the native mitochondrial enzymatic protective systems in flatworms is available, other than the report on the presence of a mitochondrial variant of TGR in *Echinococcus granulosus* larvae [14]. In the present paper we report the purification and characterization of TGR from larval *T. crassiceps* mitochondria.

## 2. Materials and Methods

### 2.1. Reagents

All buffers and substrates, as well as PMSF, EDTA, EGTA, H_2_O_2_, DTT, and BSA were obtained from Sigma-Aldrich (St Louis, Mo, USA). Trx from human, *Escherichia coli* and *Spirulina* sources were also supplied by Sigma Company. Cytosolic Trx from *T. crassiceps* was purified by following a protocol involving ionic exchange chromatography, pseudo affinity chromatography, and hydrophobic chromatography (manuscript in preparation). Trx from *Plasmodium falciparum* was a generous gift from Katja Becker (Research Center for Infectious Diseases, Germany). Auranofin was purchased from ICN Biomedicals Inc. (USA). All reagents needed for electrophoresis, including molecular weight markers were obtained from BioRad. All chemicals were used without further purification.

### 2.2. Growth of *T. crassiceps* Cysticerci

Female Balb/c mice were inoculated by the injection of about 15 cysticerci of the *T. crassiceps* HYG strain into the peritoneal cavity, as previously described [17]. Six to eight months later, cysticerci were recovered from the peritoneal cavity. Before use, larvae were washed thoroughly with phosphate buffered saline solution (PBS).

### 2.3. Obtention of the Mitochondrial Fraction

Cysticerci were suspended in mitochondrial buffer (10 mM Hepes, 250 mM sucrose, 2 mM EGTA, and 86 *μ*M PMSF) supplemented with 0.2% saponin. After a 10-minutes period incubation, cysticerci were centrifuged at 180 × g during 20 minutes. The resultant pellet, containing tegument-free cysticerci was suspended in the same buffer without saponin and subjected to mechanical homogenization in a motor-driven Teflon pestle. The homogenate was centrifuged at 180 × g for 20 minutes, and the resultant supernatant was then centrifuged at 14600 × g for 15 minutes. The pellet, containing mitochondria, was suspended in the mitochondrial buffer and washed three times. The last pellet was suspended in a 2 : 1 ratio (v/w) hypotonic solution and stored at −45°C. Mitochondrial purity was determined using two marker enzymes, lactate dehydrogenase (cytosol), and succinate dehydrogenase (mitochondria).

### 2.4. Enzyme Assays

TrxR activity. The determination of trxR activity was performed by either of the two methods described below.

(a) DTNB reduction. This assay is based on the NADPH-dependent reduction of the artificial substrate DTNB. The reaction mixture contained, in a final volume of 0.6 mL, 100 *μ*M NADPH, DTNB at different concentrations, and 1 mM EDTA in 100 mM Tris/HCl buffer (pH 7.8). The enzyme was incubated in the presence of DTNB for 3 minutes in order to obtain the baseline. Then, a small NADPH aliquot was added and the increase in absorbance was followed spectrophotometrically at 412 nm. A value of 13.6 mM^−1^ cm^−1^ for the extinction coefficient of TNB was used in the calculations.

(b) Trx reduction. In the second method, the steady state NADPH-dependent reduction of the natural substrate Trx was followed. An enzyme aliquot was incubated in the presence of 100 *μ*M NADPH and Trx (between 0.35 and 8.8 *μ*M) in 100 mM Tris/HCl buffer (pH 7.8) containing 1 mM EDTA. After stabilization of the base line, the steady state reaction was started by adding insulin at a final concentration of 180 *μ*M and the NADPH oxidation was followed at 340 nm. The final volume of the reaction mixture was 0.12 mL. A value of 6.2 mM^−1^ cm^−1^ for the extinction coefficient of NADPH was used in the calculations.

GR activity. The reduction of GSSG was determined spectrophotometrically by following the oxidation of NADPH at 340 nm. The reaction mixture contained, in a final volume of 0.6 mL, 100 *μ*M NADPH, GSSG at different concentrations, and 1 mM EDTA in 100 mM Tris/HCl buffer (pH 7.8). The enzyme was incubated in the presence of NADPH for 2 minutes in order to obtain the baseline and the reaction was started by adding GSSG.

Hydroperoxide reductase activity. The ability of the enzyme to reduce hydroperoxide was determined by following the oxidation of NADPH in the presence of variable concentrations of H_2_O_2_. The reaction mixture contained, in a final volume of 0.6 mL, 100 *μ*M NADPH, H_2_O_2_ at the indicated concentrations, and 15 nM enzyme in 100 mM Tris/HCl buffer (pH 7.8) containing 1 mM EDTA.

Thioredoxin peroxidase activity. The ability of mitochondrial TGR to catalyze the Trx-dependent reduction of H_2_O_2_ was tested by mixing 200 *μ*M H_2_O_2_, 20 *μ*M human Trx, and 100 *μ*M NADPH in 0.5 mL of 100 mM Tris/HCl buffer (pH 7.8) containing 1 mM EDTA. The reaction was started by adding TGR at a final concentration of 15 nM and the NADPH consumption was followed at 340 nm.

Glutaredoxin activity. For the glutaredoxin activity assay of mTGR, its ability to perform thiol/disulfide exchange was determined according to Holmgren and *Ǻ*slund [18]. Reduced glutathione (GSH) was mixed with hydroxyethyl disulfide (HED) in 100 mM Tris/HCl buffer (pH 7.8) containing 1 mM EDTA and incubated for 2 minutes. Then, a small enzyme aliquot was added and the reaction was allowed to proceed for an additional minute. The amount of GSSG produced was measured by adding a small aliquot containing NADPH and yeast glutathione reductase.

Inhibition assays. The protocol followed to test the effect of auranofin on mTGR activity was as follows: an enzyme sample was incubated during 3 minutes in 0.1 M Tris/HCl buffer (pH 7.8) containing 1 mM EDTA in the presence of 100 *μ*M NADPH and the corresponding auranofin concentration. Then, either GSSG or DTNB was added to start the reaction and the oxidation of NADPH was followed spectrophotometrically at 340 nm.

The effect of calcium on TGR activity was determined by using the GSSG reductase assay. Either cytosolic or mitochondrial TGR was incubated for 3 minutes in 100 mM Tris/HCl buffer (pH 7.8) in the presence of 100 *μ*M NADPH and different concentrations of CaCl_2_. Then, the reaction was started by adding GSSG at a final concentration of 67 *μ*M.

All activity assays were carried out with fresh enzyme with no more than ten days old after purification. In all cases, an enzyme unit is defined as the amount of enzyme necessary to oxidize one *μ*mol of NADPH per minute at 25°C. Kinetic parameters were obtained by fitting the Michaelis-Menten rate equation to experimental data through Sigma Plot.

Glutamine synthetase protection assay. The ability of mTGR to protect glutamine synthetase from a thiol metal-catalyzed oxidation system was performed essentially as previously described [19], with minor modifications. The inactivation mixture contained, in a final 50 *μ*L volume, 0.15 *μ*M *Escherichia coli* glutamine synthetase, 3 *μ*M FeCl_3_, 100 *μ*M NADPH, and 10 mM DTT in 50 mM Hepes buffer (pH 7) either in the presence or in the absence of mTGR. After 15 minutes of incubation at 30°C, the residual activity of glutamine synthetase was determined by adding 1 mL of the *γ*-glutamyl transferase assay mixture (0.4 mM ADP, 0.15 M glutamine, 10 mM KH_2_AsO_4_, 20 mM NH_2_OH, and 0.4 mM MnCl_2_ in 100 mM Hepes buffer, pH 7.4) and the resultant solution was incubated for additional 30 minutes at the same temperature. Then, the reaction was stopped by adding 0.25 mL of stop mixture (33 g FeCl_3_, 20 g trichloroacetic acid, and 21 mL 11.6 M HCl per liter), and the formation of the *γ*-glutamylhydroxamate-Fe ^3+^ complex was measured at 540 nm. 

### 2.5. Electrophoresis

Polyacrylamide gel electrophoresis under denaturing conditions was performed essentially as previously described [20]. Molecular weight markers were run in parallel to estimate the subunit molecular weight of the enzyme.

### 2.6. Protein Determination

A variant of the dye-binding technique of the Lowry method was used to determine protein concentration [21]. Bovine serum albumin was used as the standard. The concentration of the albumin stock solution was determined by reading its absorbance at 278 nm (*ε* ^278^ = 6.58 for a 10 mg mL^−1^ solution). Protein concentration of different Trx stock solutions was determined by reading the absorbance at 280 nm and the use of the corresponding extinction coefficient at this wavelength (human trx: 6.8 mM^−1^ cm^−1^; *Plasmodium falciparum* trx: 11.7 mM^−1^ cm^−1^; and *Escherichia coli trx*: 11.7 mM^−1^ cm^−1^). For both *Spirulina* and *T. crassiceps* Trx, their concentration was determined by densitometry. Briefly, protein samples in increasing amounts were analyzed by PAGE under denaturing and reducing conditions. Afterwards, the gels were stained and destained by conventional procedures, and the intensity of the bands was determined by IMAGEN. The amount of protein in the *Spirulina* and *T. crassiceps* samples was estimated with help of a calibration curve obtained with an *E. coli* Trx sample run in parallel.

### 2.7. Tandem Mass Spectrometry

The protein band was excised from the Coomassie stained SDS gel, destained, reduced, carbamidomethylated, and digested with modified porcine trypsin. Peptide mass spectrometric analysis was carried out using a 3200 Q TRAP hybrid tandem mass spectrometer (Applied Biosystems/MDS Sciex, Concord, ON, Canada), equipped with a nanoelectrospray ion source (NanoSpray II) and a MicrolonSpray II head as described [22]. Spectra were acquired in automated mode using Information Dependent Acquisition. The fragment ions generated were captured and mass analyzed in the Q3 linear ion trap. Database searching and protein identification were performed from the MS/MS spectra using the Mascot Software (http://www.matrixscience.com). Mass tolerances of 0.5 Da for the precursor and 0.3 Da for the fragment ion masses with the taxonomy set to other metazoa were used.

### 2.8. Purification of TGR from *T. crassiceps* Mitochondria

A frozen mitochondrial suspension was thawed and subjected to ultrasonic treatment. Broken mitochondria were then centrifuged at 269,000 × g during 45 minutes and the resultant supernatant was adsorbed on a DEAE-sephacel column (2 × 1.6 cm) previously equilibrated in 50 mM Tris/HCl buffer (pH 7.8) containing 1 mM EDTA. After washing the column, the enzyme was eluted by an NaCl linear concentration gradient (0 to 0.5 M) prepared in the same buffer solution. Fractions containing both GR and TrxR activity were pooled, concentrated in centricon tubes to a minimal volume, and dialyzed against 5 mM sodium phosphate buffer (pH 7). The retentate was then adsorbed on a hydroxyapatite chromatography column (2.8 × 2 cm) previously equilibrated in the same solution. After washing the column, adsorbed proteins were eluted with a sodium phosphate linear concentration gradient (5 to 500 mM). Active fractions were pooled and concentrated to a minimal volume as above. The resultant solution was adsorbed on a Cibacron blue chromatography column (2.6 × 2 cm) previously equilibrated in 10 mM Tris/HCl buffer (pH 7.8). After washing the column, the enzyme was recovered by applying a 200 *μ*M NADPH pulse. Active fractions were pooled, concentrated to a minimal volume, and dialyzed against 10 mM Tris/HCl buffer (pH 7.8). The retentate was stored at −20°C.

### 2.9. Purification of Cytosolic TGR from *T. crassiceps*

The protocol followed in the purification of cytosolic TGR has been described elsewhere [15].

## 3. Results

### 3.1. Purification of Mitochondrial TGR

The protocol followed in the present work for the isolation of mitochondria from *T. crassiceps* cysticerci results in a fraction which is essentially free from other subcellular organelles, as revealed by microscopic analysis [17]. However, due to the presence of TGR in both cytosolic and mitochondrial compartments, it was necessary to check the purity of the mitochondrial preparation regarding the cytosolic fraction. To this end, the activity of the marker enzymes lactate dehydrogenase (cytosol) and succinate dehydrogenase (mitochondria) was determined. Results revealed that a majority of the total succinate dehydrogenase activity was located in the mitochondrial fraction (80 ± 6.3%). In this same fraction, barely 0.7 ± 0.16% of the lactate dehydrogenase activity was detected. This preparation was then used to purify mTGR.

**Figure 1:**
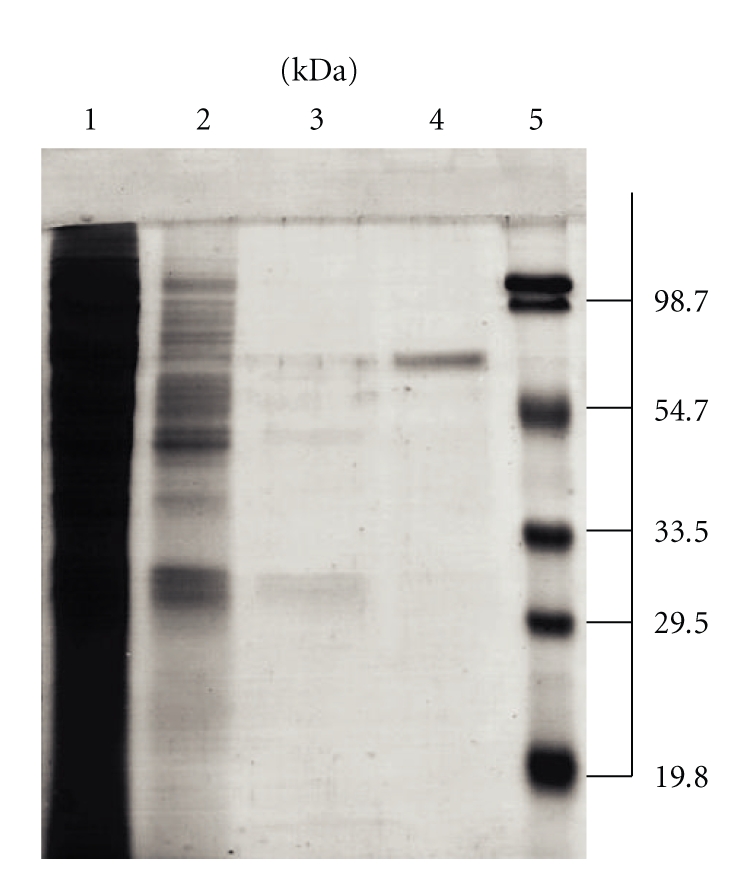
Electrophoretic patterns obtained in the purification of mTGR. Protein samples from the various steps of the purification protocol were incubated in the presence of 1% (p/v) SDS and 3% (v/v) *β*-mercaptoethanol and subjected to denaturing electrophoresis in 12% polyacrylamide gels containing 1% SDS. After run, gels were stained and destained by conventional procedures. Lanes are as follows: (1) crude extract; (2) DEAE-sephacel anion exchange chromatography; (3) Hydroxyapatite chromatography; (4) Cibacron-Blue affinity chromatography; and (5) molecular weight markers.

A summary of a typical purification procedure is shown in Table 1. With either GSSG or DTNB as substrates, enzyme activity was significantly increased throughout the purification. In the elution profiles obtained from the three chromatographic steps, the position of the GSSG- and DTNB-reductase activity peaks was coincident (data not shown). Furthermore, through the purification procedure the ratio of reductase activities remained essentially constant, suggesting both activities are located in the same protein. No evidence for additional GR or TrxR activities was found. The yield of the purification is in the range obtained for other TrxRs [9, 23]. The electrophoretic pattern revealed a homogeneous preparation in agreement with a single protein band having a molecular mass of about 65 kDa (Figure 1). This value is in the range of those reported for cytosolic TGRs [14, 15] and is higher than the subunit molecular mass of typical TrxR from animal sources, which ranged around 55 kDa [7, 9]. The identity of our preparation was confirmed by mass spectrometry. The amino acid sequence of five polypeptide fragments, comprising a total of 51 residues, showed 100% identity when compared with the corresponding fragments of *E. granulosus* mitochondrial TGR (data not shown).

### 3.2. Kinetic Properties and Specificity

**Figure 2:**
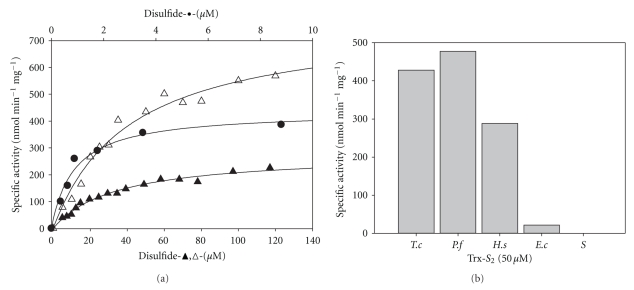
Disulfide reductase activities and specificity of mTGR. Enzyme activities were carried out at 25°C as described under Materials and Methods. (a) Saturation kinetics with different disulfides. Initial velocity data were fitted to a hyperbolic saturation kinetic function with the aid of Sigma plot. The scale of the lower abscissa represent concentration of either GSSG or DTNB, while the upper-abscissa scale corresponds to Trx concentration. (▲) GSSG reductase activity; (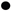) Trx reductase activity with *T. crassiceps* Trx; (Δ) Trx reductase activity with DTNB. (b) Specificity of mTGR toward exogenous Trx. The reductase activity of mTGR was assayed in the presence of a variety of prokaryotic and eukaryotic Trx. A final concentration of 50 *μ*M of the corresponding Trx was used. Abbreviations are as follows. *Tc*, *Taenia crassiceps* Trx; *Pf*: *Plasmodium falciparum* Trx; *Hs*, *Homo sapiens* Trx; *Ec*: *Escherichia coli* Trx; S, *Spirulina sp* Trx.

**Figure 3:**
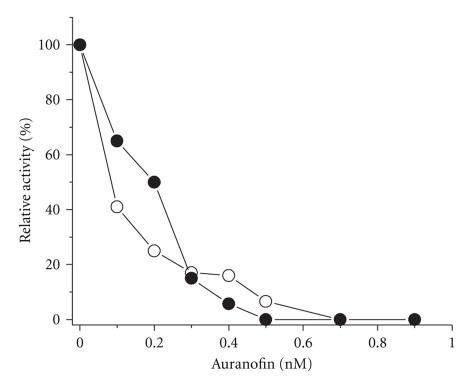
Effect of auranofin on the disulfide reductase activities of mitochondrial TGR. An enzyme aliquot (about 25 ng) was preincubated at 25°C in the presence of 100 *μ*M NADPH and inhibitor as described under Materials and Methods. To start the reaction either GSSG or DTNB was added. Final concentrations of the disulfide substrate were 40 *μ*M GSSG (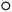); and 1 mM DTNB (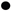).

Enzyme activity of the purified mTGR was stable up to 15 days when stored at −20°C. Interestingly, mTGR from *T. crassiceps* was less stable on storage as compared with its cytosolic counterpart. Figure 2a shows the dependence of enzyme activity on disulfide concentration with Trx, DTNB, or GSSG as substrates at 25°C. The enzyme reduced the three disulfides, albeit the physiological Trx and GSSG are clearly the preferred ones. Hyperbolic saturation kinetics were observed for the three substrates, and the kinetic constants were obtained by fitting the Michaelis-Menten equation to the data (Table 2). Mitochondrial TGR from larval *T. crassiceps* reached its maximal catalytic efficiency with endogenous Trx (5.4 × 10^5^ M^−1^ s^−1^). The ability of the enzyme to reduce GSSG is about twenty times smaller (2.2 × 10^4^ M^−1^ s^−1^). When compared with cytosolic TGR, it was evident that the cytosolic enzyme was over one order of magnitude more efficient than the mitochondrial isoform with either disulfide. Such difference is due primarily to the low turnover number of the mitochondrial enzyme. In addition to the disulfide reductase activities, mTGR also showed glutaredoxin activity. Specific activity, with HED as substrate, was 2.24 U mg^−1^. In regard of substrate specificity, It has been showed that eukaryotic TrxR has the ability to reduce Trx from a variety of sources, both prokaryotic and eukaryotic, although with a smaller catalytic efficiency when compared with the native Trx. Figure 2(b) shows the results obtained when mTGR from *T. crassiceps* was assayed for its ability to reduce exogenous Trx. Clearly, Trxs from eukaryotic origin (*Plasmodium falciparum* or human) are good substrates of the enzyme, while Trxs from bacterial sources (*E. coli* or *Spirulina*) were not recognized at the concentrations tested. The reductase activities of mTGR from *T. crassiceps*, with either GSSG or DTNB as substrates, were inhibited by the gold compound auranofin (Figure 3). Nanomolar concentrations of the latter were required to achieve a full inhibition of the enzyme activities, suggesting that mTGR depends on an essential selenocysteine residue for activity.

**Figure 4:**
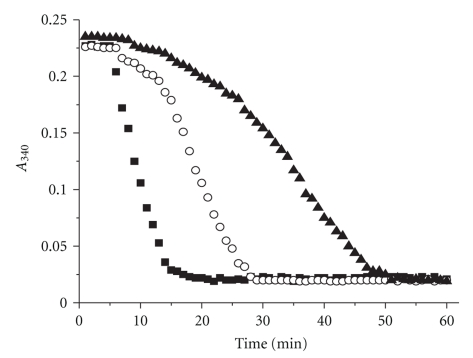
Full time courses of the GSSG reductase activity of mTGR. Enzyme was incubated at 39°C in the presence of 40 *μ*M NADPH; then, reaction was started by adding a GSSG aliquot. Final concentrations of GSSG were (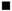) 80 *μ*M; (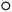) 500 *μ*M; and (▲) 1 mM.

Like cytosolic TGR from *E. granulosus *and *T. crassiceps* [15, 24], the mitochondrial enzyme showed hysteresis (Figure 4), with the appearance of a lag time in the enzymatic assays when GSSG is used as substrate at moderate or high concentrations. The magnitude of the lag time was independent of the preincubation of the enzyme with either NADPH or GSSG. However, the presence of micromolar concentrations of either Trx or GSH in the assay mixture resulted in a significant reduction in the magnitude of the lag time, as reported for cytosolic TGR from *T. crassiceps* and *E. granulosus* [15, 24].

### 3.3. Dependence of Enzyme Activity on pH and Temperature

**Figure 5:**
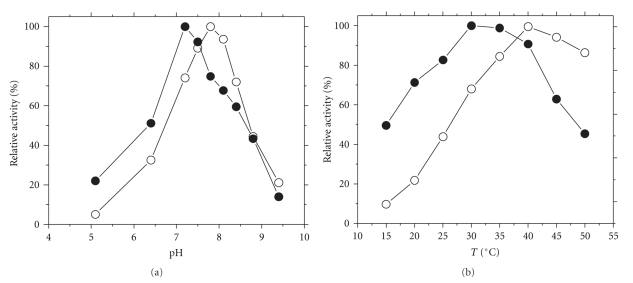
Dependence of the reductase activities of mTGR on pH and temperature. Enzyme assays were carried out as described under Materials and Methods by varying either pH at 25°C (a) or temperature at pH 7.8 (b) using either 1 mM DTNB (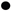) or 60 *μ*M GSSG (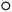) as substrate and 100 *μ*M NADPH. Each point represents the average of two independent experiments.

The effect of pH and temperature on the reductase activities of mTGR is shown in Figure 5. Interestingly, the optimum pH for DTNB and GSSG was not coincident. Thus, while for the GR activity the highest value is located at pH 7.8, for the DTNB reductase activity the optimum pH is 7.2 (Figure 5(a)). With temperature a similar pattern was observed (Figure 5(b)). It is noteworthy that the optimum value with GSSG (40°C) is almost coincident with the body temperature of the intermediary host of *T. crassiceps* (39°C). From the dependence of enzyme activity on temperature at the rising portion of the curve, it was possible to determine the energy change associated with the activation step for both substrates. Values of 57.8 ± 1.6 kJ mol^−1^ and 29.5 ± 5.4 kJ mol^−1^ were obtained for the activation energy with GSSG and DTNB, respectively, from the slope of the corresponding Arrhenius plot.

### 3.4. Hydroperoxide Reductase Activity of mTGR

**Figure 6:**
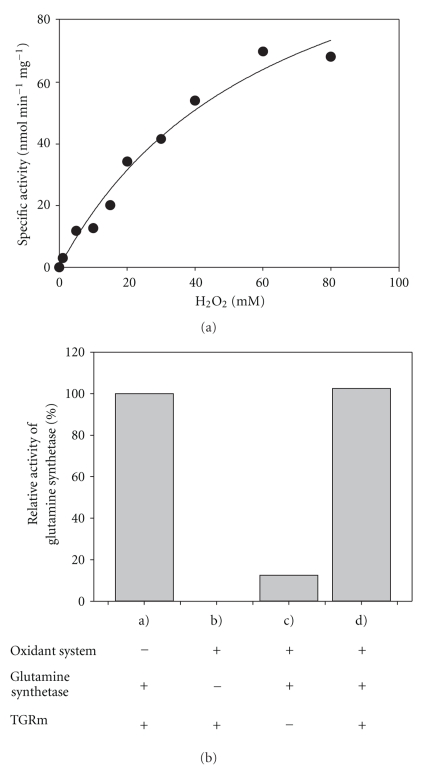
Antioxidant and protective activities of mTGR. (a) H_2_O_2_ reductase activity. The NADPH-dependent reduction of H_2_O_2_ by mTGR was assayed at 25°C in the presence of 100 *μ*M NADPH. (b) Protection of glutamine synthetase. A mTGR aliquot (10 nM) was incubated along *E. coli* glutamine synthetase (150 nM) in the presence of a mixed-function oxidation system in a final volume of 50 *μ*L. After 10 minutes, 2 mL of the *γ*-glutamyltransferase assay mixture was added. Additional details are described under Materials and Methods. (a) positive control; (b) negative control; (c) Full mixture without mTGR; and (d) Full mixture.

 It has been reported that TrxR from mammalian sources [8], as well as TGR from *Schistosoma mansoni* [13] catalyze the NADPH-dependent reduction of hydroperoxide compounds, albeit with a low efficiency. Figure 6(a) shows the activity of mTGR with H_2_O_2_ as substrate at a constant concentration of NADPH (100 *μ*M). Although H_2_O_2_ was recognized as a substrate by the enzyme, concentrations of H_2_O_2_ in the milimolar range were needed to obtain measurable velocities. Above 80 mM H_2_O_2_, a strong inhibitory effect was observed. Fitting the Michaelis-Menten equation to the data gave an apparent K_m_ value of 64 mM and a catalytic efficiency value of 2 s^−1^ M^−1^. Interestingly, when Trx was included in the assay mixture, a steady state turnover of H_2_O_2_ consumption was observed, even at micromolar levels of the peroxide. Under the experimental conditions used, a specific activity of 304 mU mg^−1^ was obtained.

### 3.5. Protective Competence of mTGR against Oxidative Damage

Because *T. crassiceps* mitochondria have the potential to produce H_2_O_2_ in significant amounts [16], we decided to test the ability of mTGR to directly protect *E. coli* glutamine synthetase against enzyme inactivation by a DTT/Fe^3+^/O_2_ system [19]. When glutamine synthetase was preincubated in the presence of the thiol/Fe^3+^/O_2_ mixed-function oxidase system, mTGR fully protected the enzyme activity from oxidative inactivation (Figure 6(b)). In contrast, when mTGR was omitted from the preincubation mixture, glutamine synthetase activity was not detected.

### 3.6. Inhibition by Calcium Ions

**Figure 7:**
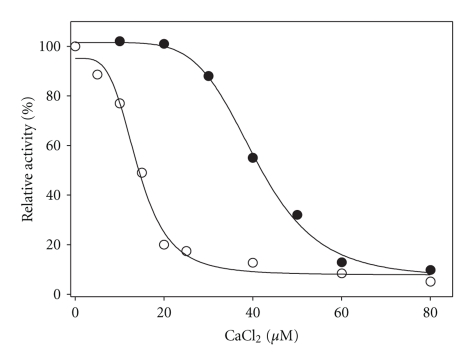
Effect of calcium on cytosolic and mitochondrial TGR. The incubation protocol is described in detail under Materials and Methods. Solid lines represent the fitting of experimental data to the equation described in the Results section. Each point represents the average of three independent determinations. (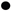) mitochondrial TGR; (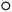) cytosolic TGR.

Figure 7 shows the effect of micromolar concentrations of calcium on the GSSG reductase activity of cytosolic and mitochondrial TGR. A differential effect is clearly evident, such that the cytosolic variant showed a higher sensitivity to calcium ions. Further, from the slope of the traces in the transition region, a higher degree in cooperativity for the mitochondrial enzyme is apparent. In order to obtain the inhibitor concentration causing 50% inhibition (IC_50_), as well as a measure of the cooperativity, data were fitted to the following equation:

(1) $$y=\min\;+\frac{\max\;-\min\;}{1+{\left(x/\text{I}{\text{C}}_{50}\right)}^{n}},$$

where “*y*” represents the residual activity, “max” and “min” the control and fully inhibited velocity data, respectively, “*x*” corresponds to the calcium concentration and *n* is the Hill coefficient. For mitochondrial TGR, values of 40.7 ± 0.8 *μ*M and 5.7 ± 0.6 for IC_50_ and *n,* respectively, were obtained. The corresponding values for the cytosolic variant were 14 ± 0.7 *μ*M and 4 ± 0.6. 

## 4. Discussion

Gene fusion resulting in polypeptide products with more than one enzyme activity has been demonstrated in parasitic protozoa. In these unicellular eukaryotes, the first two enzymes of the pentose phosphate pathway (glucose 6-phosphate dehydrogenase and 6-phosphogluconolactonase), as well as the enzymes involved in thymidylate biosynthesis (dihydrofolate reductase and thymidylate synthase) are located in bifunctional proteins [25–27]. In parasitic flatworms, TGR appear to represent an additional example of this strategy. In these organisms, fusion of the Grx and TrxR modules has resulted in a multifunctional enzyme with GR and TrxR activities, as well as the ability to perform thiol/disulfide exchanges. To date, cytosolic TGRs from the adult stage of the blood fluke *S. mansoni* [13] and from larval *T. crassiceps* [15] have been purified and characterized. However, regarding the mitochondrial isoform of the enzyme no information is available, other than the demonstration of its potential presence in *E. granulosus* protoscoleces [14].

The results shown in the present work indicate that mitochondria from the larval stage of *T. crassiceps* contain a functional TGR, which is involved in the maintenance of the reduced forms of both glutathione and thioredoxin. Thus, like in the cytosol, a single reductase is present in mitochondria. The full inhibition of the disulfide reductase activities of mTGR by nanomolar concentrations of auranofin suggests a critical dependence on a selenocysteine residue for its function. This observation is reinforced by the demonstration of a significant thioredoxin peroxidase activity of the enzyme. In this sense, it has been shown that selenocysteine plays an essential role in such activity. A TrxR mutant lacking such residue was unable to exhibit peroxidase activity, but the latter was fully restored by adding selenocysteine at micromolar levels [8]. Thus, similar to its cytosolic counterpart [13–15], mTGR depends on selenocysteine for enzyme activity. Such situation is in contrast to that observed for TrxR from dipteran insects or nematodes. In *Drosophila melanogaster*, both cytosolic and mitochondrial TrxRs depend on a disulfide redox center located at their C-terminal end [28]. In contrast, in the free-living nematode *Caenorhabditis elegans* an unusual situation was found. In this organism, the cytosolic variant of TrxR depends on Sec for activity, while in the mitochondrial TrxR, Sec has been replaced by cysteine [29].

It has been shown recently that auranofin is an irreversible inhibitor of *S. mansoni* TGR [30]. Such observation is in agreement with the low concentration of the gold compound required in the present work to achieve a full inhibition of the disulfide reductase activities of *T. crassiceps* mTGR. Interestingly, *S. mansoni* TGR requires a relatively long incubation time (15 minutes) in the presence of both auranofin and NADPH to obtain a significant degree of inhibition [30], but mTGR from *T. crassiceps* was clearly inhibited within 3 minutes of incubation. Such difference in the response of the *S. mansoni* and *T. crassiceps* enzymes could be explained by different kinetic reactivities toward the gold compound. In this sense, selenocysteine appears to play a catalytic role in the inhibition process by mediating the transfer of gold from auranofin to the enzyme [30]. This point deserves further insight.

On the other hand, the notable inhibitory ability of auranofin on the disulfide reductase activities of either mitochondrial or cytosolic TGR, clearly points to a potential therapeutic use of such gold compound for the control of cysticercosis. In this sense, data from our laboratory revealed a high susceptibility of *T. crassiceps* larvae to auranofin. After a 12-hours period in the presence of 10 *μ*M auranofin, a full mortality was observed (manuscript in press). 

Although TGR is a multifunctional enzyme, its catalytic efficiency with Trx is higher than with GSSG. In Table 3 the catalytic efficiencies of TGR from various sources for GSSG and Trx are compared. In all cases, the enzyme shows a clear preference toward Trx. With the exception of cytosolic TGR from *T. crassiceps*, the catalytic efficiency of the enzymes with Trx is at least one order of magnitude higher than with GSSG. The low Trx/GSSG ratio of 3.3 obtained for the cytosolic variant of TGR from *T. crassiceps* can be the result of using Trx from *P. falciparum* as substrate. On the other hand, when compared with animal TrxR, the catalytic efficiency of cytosolic TGR, with endogenous Trx as substrate, is in the same range (1 to 6 × 10^6^ s^−1^ M^−1^). However, a very different situation is found with GSSG. For typical GR from various sources, catalytic efficiency values are in the range of 1 to 4 × 10^6^ s^−1^ M^−1^, an order of magnitude higher than the corresponding value for any characterized TGR (Table 3). Although the relatively low catalytic efficiency of TGR toward GSSG has been partially compensated by relatively low *K*_m_ values, such strategy is far from effective. Thus, the incorporation of both disulfide reductase activities in a single multifunctional enzyme has resulted in a significant loss of catalytic efficiency with one of the disulfide substrates. Regarding the ability of the enzyme to reduce exogenous Trx, it is interesting to note that, like typical TrxR from animal sources, mTGR from *T. crassiceps* recognizes Trx from eukaryotic origin. In contrast, bacterial Trx is a poor substrate. Such apparent inability to reduce prokaryotic Trx can be, however, the result of very high K_m_ values. In this sense, it has been reported that mTrxR from the nematode *C. elegans* is able to reduce bacterial Trx, albeit high concentrations of such substrate are needed [29].

 An interesting finding regarding mTGR was the pH dependence of the reductase activities. Although in both cases an optimal pH value was clearly defined, 7.8 for GSSG and 7.2 for DTNB, the position of such optima was not coincident. Such results suggest that in the reduction pathway of GSSG and DTNB, different acid and/or basic groups, with different pK_a_ values, are involved. In this sense, it has been shown that the GSSG reducing activity of TGR depends on the Grx domain. A deletion mutant of *E. granulosus* TGR, lacking the Grx domain, was unable to catalyze the reduction of GSSG, but the DTNB-reduction was unaffected [24]. Hence, a dissociable group located on the glutarredoxin domain, involved in the binding and/or reduction of GSSG, could explain the discrepancy in the pH profiles of the GSSG- and DTNB reductase activities of mTGR. Alternatively, the preferential binding of either GSSG or DTNB for a different ionization state of the same functional group would result in a similar dependence on pH of the two reductase activities. Further work is needed in order to clarify this point.

Regarding the effect of temperature on the enzymatic activities of mTGR, it was also observed different optimal values for GSSG or DTNB as substrates. However, the most interesting finding was the coincidence of the optimal temperature of the GSSG reductase activity (40°C) with the body temperature of the intermediary host of *T. crassiceps* (39°C). Such observation reveals that, in regard to the GSSG reductase, mTGR is working under optimal thermal conditions in vivo. The thermodynamic analysis shows a higher dependence on temperature for the GSSG reductase activity as compared with the DTNB reductase activity. Unfortunately, no similar study has been performed with any TGR, thus preventing any comparison.

On the other hand, mTGR was able to reduce H_2_O_2_ in a NADPH-dependent manner, albeit with low catalytic efficiency. This observation is in accordance with previous reports showing H_2_O_2_ reductase activity for the *S. mansoni* TGR and mammalian TrxR [8, 13]. Although catalase in mammals represents the primary enzyme defense against H_2_O_2_ generated in mitochondria, there are no reports on the existence of such enzyme in parasitic flatworms. Catalase activity was not detected in *S. mansoni* [31]. Interestingly, mTGR from *T. crassiceps* cysticerci showed a significant thioredoxin peroxidase activity. Although a detailed kinetic study has not been performed, the specific activity obtained of 304 mU mg^−1^is in the range reported for recombinant peroxiredoxin from the liver fluke *Fasciola hepatica* (1200 mU mg^−1^) [32] and for mammalian peroxiredoxin (4000 mU mg^−1^) [33]. Thus, the possibility that TGR is acting as a defense system in *T. crassiceps* mitochondria is open. The finding that mTGR has a significant thioredoxin peroxidase activity in the presence of micromolar concentrations of both trx and H_2_O_2_, as well as the full protection conferred to glutamine synthetase against oxidative inactivation, strongly support such proposal.

The higher sensitivity of cytosolic TGR to calcium, as compared with the mitochondrial counterpart TGR of *T. crassiceps* reported in this work is similar to that observed in TrxR from rat liver [34]. Such finding was an unexpected one, because in parasitic flatworms both isoforms of TGR are apparently coded by a single gene [14]. The micromolar range of calcium concentrations where the inhibition was observed open the possibility that TGR could be involved in a kind of redox regulatory mechanism, as discussed below. 

The hysteretic behavior observed at relatively high concentrations of GSSG, described for cytosolic TGR from *E. granulosus* [24] and *T. crassiceps* [15] is also present in mTGR from the latter. Thus, it can be concluded that a GSSG concentration-dependent hysteretic pattern appears to be a common feature of the multifunctional TGR. A model intended to explain such interesting kinetic behavior has been recently proposed [24]. Such proposal was based on glutathionylation of specific cysteine residues of the enzyme located on the thioredoxin reductase moiety of TGR. However, no detailed kinetic study is yet available. The recent determination of the three-dimensional structure of *S. mansoni* TGR [35] will help to obtain a thorough understanding of this peculiar kinetic pattern. It is tempting to speculate on the potential physiological significance of both the hysteretic behavior and calcium inhibition of TGR. The essential role that Trx plays in the maintenance of the reduced state of the sulphydryl groups in a variety of intracellular proteins has been demonstrated [36, 37]. This ability is clearly dependent on a fully active TrxR. Thus, inhibition of the disulfide reductase activities of TGR due to an increase in either calcium or GSSG would result in a transient disturbance in the dithiol/disulfide equilibrium of proteins. Such disturbance could potentially change the activity of a number of essential protein factors that may be critical to initiate signals that control a variety of cell process. Clearly, more work is needed in order to elucidate such possibility.

Finally, it must be noted that, albeit in parasitic flatworms both cytosolic and mitochondrial TGRs are coded by a single gene, some data strongly suggest its mature functional state are different. Thus, as shown in Table 3, the catalytic efficiency of the cytosolic variant, with either Trx or GSSG as substrate, is significantly higher as compared with its mitochondrial counterpart. Furthermore, the stability of both isoforms on storage as well as its sensitivity to calcium is clearly different. A plausible explanation of this observation could be found in a different oxidative stress between the cytosolic and mitochondrial compartments. As noted in the introductory section, mitochondrion is the site where much of the reactive oxygen species are produced, resulting in a more oxidative environment as contrasted with cytosol. Thus, disulfide formation in mitochondrial proteins could be induced by mild oxidation, resulting in covalently modified forms with a conformation different enough for it to alter some functional properties. Examples of such phenomena have been described already [38, 39].

## Figures and Tables

**Table 1:** Summary of the mTGR purification procedure.

Fraction	Vol_Total_ (ml)	Protein (mg)	Specific Activity (mU*/mg)			Activity ratio	Activity (mU)	Purication (fold)	Yield (%)
			GSSG	DTNB	HED	DTNB/GSSG		DTNB	

Crude extract	13.0	33.5	11.7	35,0	128.5	3.0	1172.5	1.0	100
DEAE-Sephacel	3.3	9.37	34.0	75.2	138.0	22	704.6	2.1	60
HA-Ultrogel	3.2	2.23	38.7	184.0	532.1	4.7	410.3	5.3	35
Cibacron-Blue	1.72	0.186	229.0	1091.0	2242.8	4.7	202.9	31.2	17

An enzyme mU is defined as the amount of protein needed to oxidize one nmol of NADPH per minute at 25°C.

Date obtained at 25°C and pH 7.8.

**Table 2:** Kinetic constants for mTGR using different disulfides as substrates.

Disulfide		mTGR	
	K_m_	*k*_cat_	*k*_cat_ */*K_m_
	(*μ*M)	(S^−1^)	(M^−1^ S^−1^)
Trx-S_2_ **T. crassiceps**	0.87	0.47	5.4 x 10^5^
Trx-S_2_ *P. falciparum *	28.3	0.81	2.8 x 10^4^
Trx-S_2_ *H. sapiens *	21.3	0.44	2.1 x 10^4^
GSSG	41.4	0.84	2.0 x 10^4^
DTNB	34.5	0.30	8.7 x 10^3^

Date obtained using 100 *μ*M NADPH at 25°C and pH 7.8.

**Table 3:** Comparison of TGRs catalytic efficiencies using thioredoxin or glutathione as substrate.

Enzyme		*k*_cat_/K_m_ (M^−1^s^−1^)	
	Trx-S_2_	GSSG	Trx-S_2_/GSSG
TGR^a^-*Mouse testis *	1.89 × 10^6^	0.18 × 10^6^	10.7
cTGR^b^-**T. crassiceps**	1.13 × 10^6^	0.34 × 10^6^	3.3
TGR^c^-S. *Mansoni *	4.70 × 10^6^	3.0 × 10^5^	15.7
mTGR^d^-**T. crassiceps**	5.40 × 10^5^	2.2 × 10^4^	24.5

^a^Taken from reference [11] using Trx-*E*. *coli.*

^b^Taken from reference [15] using Trx *P. falciparum.*

^C^Taken from reference [13] using Trx (natural substrate).

^d^This report with Trx (natural substrate).
